# Inter-episodes earthquake migration in the Bohai-Zhangjiakou Fault Zone, North China: Insights from numerical modeling

**DOI:** 10.1371/journal.pone.0251606

**Published:** 2021-05-19

**Authors:** Bo Shao, Guiting Hou, Jun Shen

**Affiliations:** 1 Key Laboratory of Orogenic Belts and Crustal Evolution, Education Administration, School of Earth and Space Sciences, Peking University, Beijing, China; 2 Institution of Science and Technology, China Three Gorges Project Corporation, Beijing, China; 3 Institute of Disaster Prevention, Beijing, China; Chinese Academy of Geological Sciences, CHINA

## Abstract

In this paper, we focus on why intraplate seismic initiation and migration occurs, which has widely been considered to be caused by static stress triggering caused by earthquakes, as well as post-seismic slips. To illustrate the mechanism underlying large earthquakes, in particular the migration caused by two key episodes that occurred after 1500 in the Bohai-Zhangjiakou Fault Zone (BZFZ) of North China, we developed a high-resolution three-dimensional viscoelastic finite element model that includes the active faults with vertical segmentation, their periodical locking, and the lithosphere heterogeneity. We used the birth and death of element groups to simulate stress intensity changes during the two episodes (named Episode I and II), with our results showing that the Tangshan earthquake was primarily triggered by the Sanhe-Pinggu M8.0 earthquake in 1679, whereas the Zhangbei M6.2 earthquake in 1998 was not triggered by earthquakes in Episode I. According to our work, the calculated stress changes in the different segments of the fault zone correspond to the magnitude of the triggered earthquakes. Further, the largest stress decrease was near the Sanhe-Pinggu fault and occurred the largest earthquake in Episode I, whereas the largest stress increase was near the Tangshan fault and occurred during the largest earthquake in Episode II. Given the above, we propose a model for seismic migration to describe the dynamic mechanisms of earthquake migration within the BZFZ and North China, in which the factors affecting both the seismic migration path and intensity primarily include the distance between the triggered active fault and the original fault, the coupling of the active faults, the location and scale of the low-velocity anomaly, its distance from the active fault, and the location and scale of the crustal thinning.

## Introduction

Increasing amount of historical evidence regarding the intraplate earthquakes shows episodic seismicity and clustering seismic migration within some mid-continents, including North China and New Madrid [1,2]. Seismic migration has been substantially researched within the continental margins, especially in the San Andreas active fault zone and Japan [3–9]; however, unlike the mechanism of earthquakes in the continental margins, researchers disagree over the detailed mechanisms that cause intraplate seismic initiation and migration. In general, causes are assumed to be associated with pre-existing faults, stress fields, and lithospheric structures [2,10]. Further, individual faults may remain dormant for long periods of time and then become active for short periods of time, thereby resulting in episodic and spatially migrating earthquakes, because of the low tectonic loading rate exhibited by several faults within the mid-continents.

Static stress triggering caused by earthquakes, as well as post-seismic slips, has widely been considered a cause of such intraplate seismic initiation and migration [5,11,12]. Previous studies show denote that such stress changes variations are largely considerably limited with respect to the spatial distribution, horizontal segmentation, and friction coefficients of the ruptured faults [13–18]; however, the vertical segmentation and periodic locking of active faults have seldom been quantitatively considered in previous research. Therefore, in this study, we focus on these infrequently covered perspectives.

The Bohai-Zhangjiakou Fault Zone (BZFZ) underlies the capital region of China and several megalopolises. The BZFZ is an extremely active fault system and is arguably the most important intraplate seismic belt within the North China Craton since it has been dominated by moderate to strong earthquakes since 1500. Some of the largest earthquakes that have occurred include the Sanhe-Pinggu M8.0 earthquake in 1679, the Tangshan M7.8 earthquake in 1976, and the Zhangbei M6.2 earthquake in 1998 [19–24]. To illustrate the mechanisms underlying earthquake migration in North China, simulations of stress fields and fault interactions using the finite element method (FEM) have been employed, thereby addressing the active faults distribution, their horizontal segmentation, basin-range patterns, and other similar factors [13,25–32]. Conversely, the influence of velocity anomalies on the seismic dynamics in North China has only recently been considered via a conceptual model [28]. Previous studies have obtained only static modern stress fields using complex three-dimensional FEM models to explain the distribution of historical earthquakes beyond just earthquake migration along the time axis [29–31]. Further, the vertical segmentation and periodic locking of faults in North China has not been addressed in previous research.

Therefore, in our work, we used a high-resolution three-dimensional viscoelastic FEM model that considers active faults with vertical segmentation, their periodic locking, and the lithosphere heterogeneity to illustrate the mechanism of large earthquake migrations in the BZFZ between the two key episodes after 1500, i.e., the Sanhe-Pinggu M8.0 earthquake in 1679 and the Zhangbei M6.2 earthquake in 1998.

## Geologic and earthquake backgrounds

**Fig 1:**
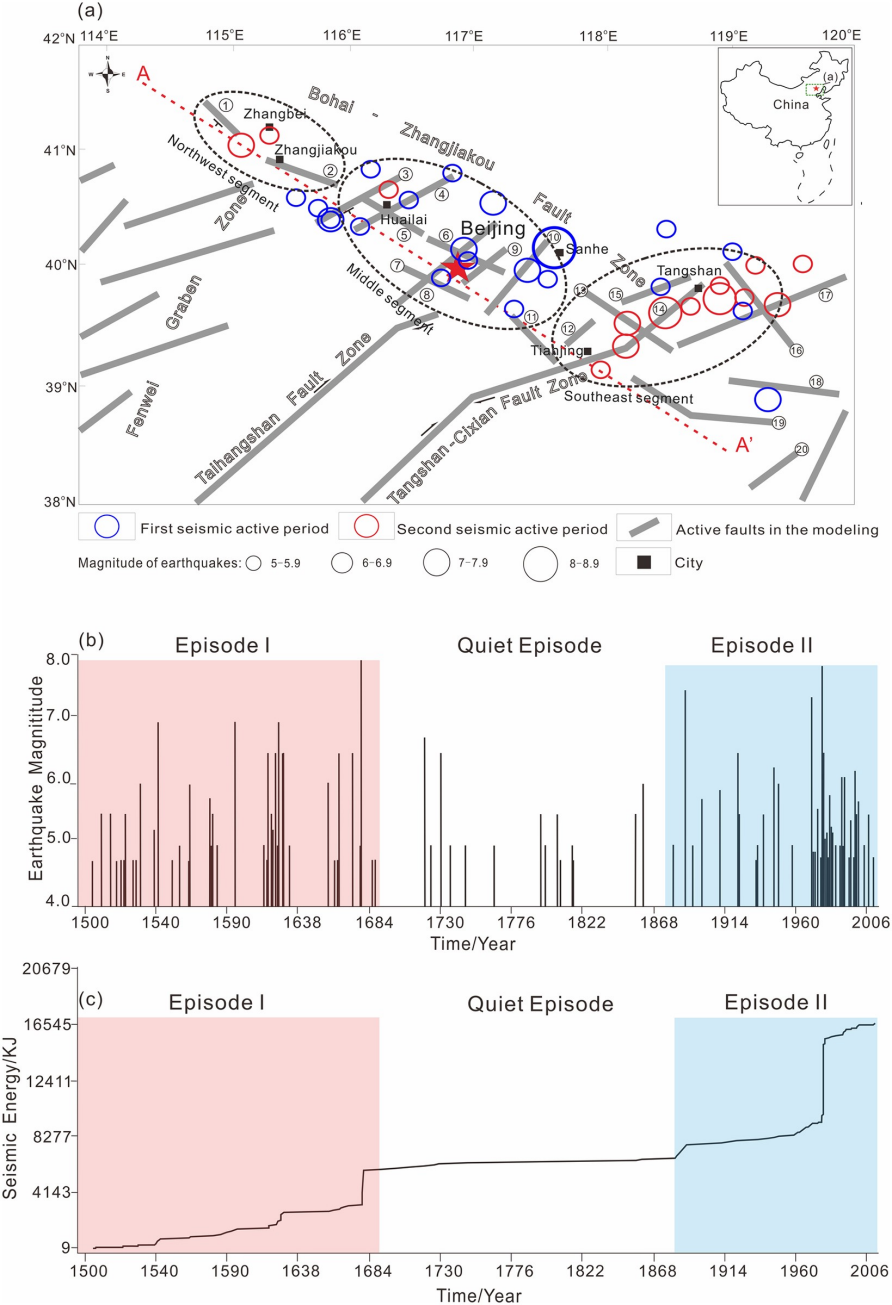
(a) Main active faults and earthquakes (M≥5) distribution in the BZFZ. (b) Occurrence and magnitude (M ≥4.5) of earthquakes in the BZFZ from 1500 to 2006 A.D. (c) The time-averaged rate of seismic energy release in two active episodes and the quiet episode. In (a), the blue circles represent earthquakes in episode I. In (b), The red circles represent earthquakes in episode II. The red part includes earthquakes in episode I. The blue part includes earthquakes in episode II. In (b), colors for episodes are correlated with those in the (b).

As shown in Fig 1(a), the BZFZ is an expansive NW-trending active fault zone that begins in northern Zhangjiakou and extends southeastward to the Bohai Sea, overall covering a total length of 700 km and a maximum width of 60 km [19,23,31,33–36]. From this figure, the BZFZ can be considered to be a series of NE-trending en échelon normal faults [37]. All of these faults appear to merge into one deep large-scale fault zone that cuts the lithosphere with increasing depth. The deep fault zone lies on the northern margin of the Cenozoic Bohai Bay Basin where volcanoes and earthquakes continue to occur [23,24,27,28,38–43].

The earthquake catalog for the BZFZ and its adjacent areas, which appears to be complete for M6 events and greater from 1500 to 1998 [44], includes at least four M7 or greater earthquakes. More specifically, the catalog shows two active episodes (i.e., Episodes I and II) and one Quiet Episode since 1500, as illustrated in Fig 1(b). In Fig 1(c), for Episodes I and II, the seismic energy in North China consistently increased in a step-type fashion. Further, in the Quiet Episode, the frequency of earthquakes was relatively low, and the seismic energy tended to be steady throughout. Mid-strong earthquakes divide the episodes. In the quiet episode, no great earthquakes (M>7) ever happened.

Most of the earthquakes in the BZFZ, especially those greater than M6.0, tended to cluster at the intersections of the NW-trending and NE-trending fault zones. Here, as shown in Fig 1(a), the fault zones are divided into the following three segments: (1) the Zhangjiakou or northwest segment, which lies at the intersection of the BZFZ and the Shanxi Rift Belt; (2) the Huailai-Beijing-Sanhe or middle segment, which lies at the intersection of the BZFZ and the Taihangshan Fault Zone; and (3) the Tangshan or southeast segment, which lies at the intersection of the BZFZ and the Tangshan-Cixian-Ninghe Fault Zone [45].

In Episode I, large earthquakes were concentrated in the middle segment, in particular the Sanhe-Pinggu M8.0 earthquake in 1679. In Episode II, large earthquakes were concentrated in the southwest and northwest segments, in particular the Tangshan M7.8 earthquake in 1976 and the Zhangbei M6.2 earthquake in 1998 [44]. Researchers agree that earthquakes migrated from the middle segment to the northwest and southeast segments between the two episodes [37,45]. The spatiotemporal occurrence of earthquakes in the BZFZ shows long-distance migrations of earthquakes within the two active episodes since 1500. Over the last two millennia, no large earthquake within the BZFZ has re-ruptured along the same fault segment. Such long-distance migrations of earthquakes cannot be entirely attributed to stress triggering from earthquake-induced changes in the Coulomb stress near the same ruptured fault segments [27,46]. Therefore, to properly research earthquake migration, we must also consider regional stress changes caused by active faults with different spatial patterns and segmentations, as well as the lithospheric heterogeneity.

## Model and methods

To study the mechanism underlying earthquake migration within the BZFZ, we developed a three-dimensional viscoelastic FEM model that includes all active faults, vertical segmentations, and lithospheric heterogeneity.

**Fig 2:**
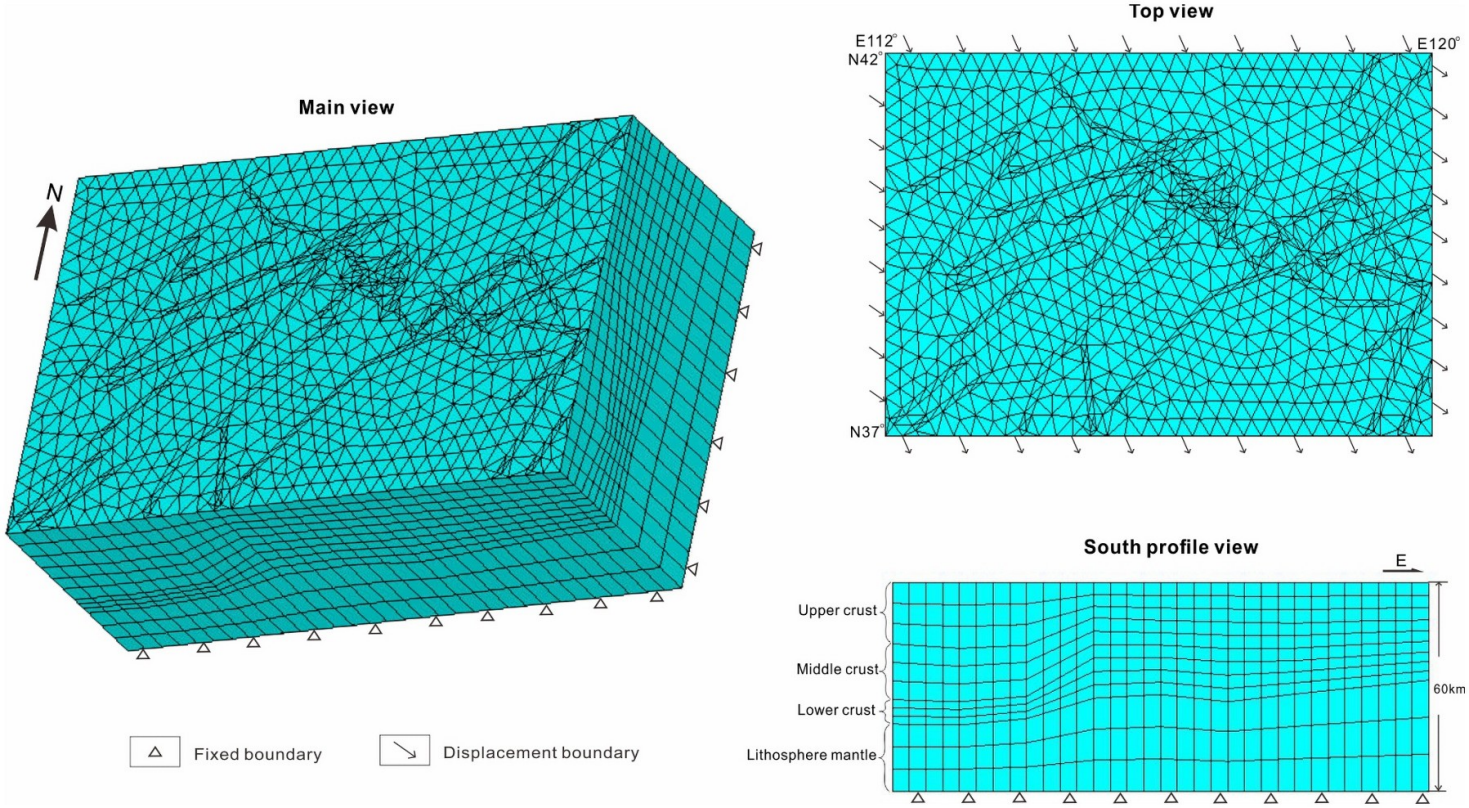
The main view, top view and south profile view of the 3D model. The model domain extends from 113°E to 120°E in longitude, from 37°N to 42°N in latitude, and covers 60km in depth. The lithosphere is modeled using a layered rheology structure, which includes four types of rocks (upper crust, middle crust, lower crust, and lithosphere mantle) (Table 1).

We constructed the FEM using the ANSYS 14.5 (University Version) finite element software package. Our model domain extends from 113°E to 120°E longitude and from 37°N to 42°N latitude, covering a depth of 100 km. As shown in Fig 2, we discretized the model using 174,056 15-node tri-prism elements in latitude × longitude × depth using a spatially variable finite-element mesh with the finest resolution of 0.5°×0.5°×3 km.

We modeled the lithosphere using a layered rheology structure that includes four types of rock; as summarized in Table 1, these rock types included upper crust, middle crust, lower crust, and part of the upper mantle. Imposed ~60 km depth of the model in eastern North China Craton [47], the depth range for upper crust, middle crust, lower crust were defined Accordance with the lithospheric structure of the Crust 1.0 [48]. Our model also accounts for the viscoelastic behavior within the lithosphere [49]. More specifically, the viscoelastic rheology is controlled by the following Maxwell constitutive equation [50]:

$$\dot{Ɛ}=\sigma /\eta_{eff}+\dot{\sigma }/E$$ (1)

where $\dot{Ɛ}$ represents the strain rate, σ is the differential stress, $\dot{\sigma }$ represents the stress rate, E is Young’s modulus reflecting the elastic component, and ηeff is the effective viscosity.

**Table 1:** The viscoelastic properties used for the model.

Layer	Facie	Rock type	*A (MPa*^*-n*^*·s*^*-1*^*)*	*n*	*E (KJ·mol*^*-1*^*)*	*ρ* (kg·m^-3^)	*T*_*0*_ (°C)	*V*_*p*_^*0*^ (km·s^-1^)	*V*_*s*_^*0*^ (km·s^-1^)	*-dV*_*p*_*/dT* (m·s^-1^°C^-1^)
**Upper crust**	greenschist	granitic	2.0×10^−4^	1.9	141	2580	200	6.1	3.55	60
**Middle crust**	amphibolite	diorite	3.8×10^−2^	2.4	219	2640	400	6.4	3.75	15–31
**Lower crust**	granulite	gabbro	8.0×10^−3^	4.2	243	2850	600	6.9	3.7	22–25
**Mantle**	dry dunite	peridotite	2.0×10^3^	4.0	471	3270	800	8.0	4.45	42–55

*Part of the data from Liu and Wu [49].

In the above viscoelastic model, Young’s modulus E and Poisson’s ratio ν can be calculated using the following equations, which essentially define Hooke’s law in three dimensions [2,51]:

$$V_{p}=\sqrt{(\lambda +2\mu )/\rho }$$ (2)

$$V_{s}=\sqrt{\mu /\rho }$$ (3)

E=μ(3λ+2μ)/(λ+μ) (4)

ν=λ/2(λ+μ) (5)

where Vp represents the P-wave velocity, Vs represents the S-wave velocity, ρ represents rock density, μ and λ are Lamé parameters, E is Young’s modulus, and ν is Poisson’s ratio.

We also assume that an approximately linear relationship exists between temperature and velocity when the pressure and rock types are given [2,50]. Since a layer structure guarantees an approximately constant pressure at each layer, we can calculate temperature via interpolation from the referenced geothermal sections with corresponding velocities. Then, the viscous properties are calculated by the Power Law for effective viscosity, η_eff_:

$$\eta_{eff}={\dot{Ɛ}}^{\frac{1-n}{n}}{\left[A\;\text{exp}(-\frac{E^{*}}{RT})\right]}^{-\frac{1}{n}}$$ (6)

Further, layer temperature T is calculated as follows:

$$T=T_{0}+(V_{p}-V_{p}^{0})/(-dV_{p}/dT)$$ (7)

where R is the universal gas constant (i.e., 8.31 J mol−1 K−1); T represents temperature; constants A, n, and E* for the four rock types are based on laboratory results, and the strain rates $\dot{Ɛ}$ are assumed as 10–15 s -1; T0 is the reference temperature for each layer, Vp0 is the reference velocity for each layer, and −(dVp)/dT is the temperature derivative of the P-wave. Finally, the time step is 10,000 years, with the initial geometry summarized in Fig 2.

**Fig 3:**
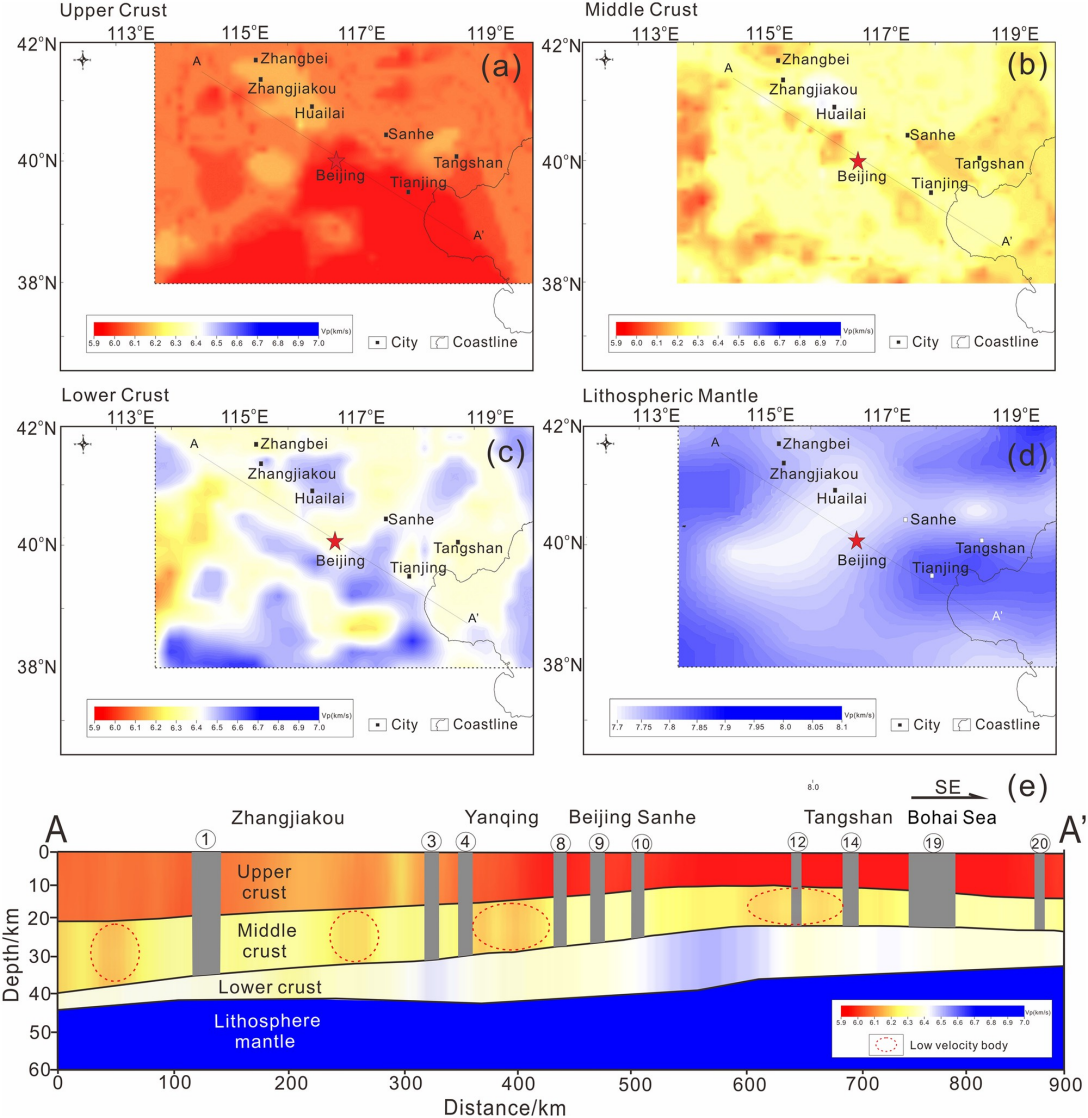
P wave velocity structure in the research zone. (a) P wave velocity structure of the Upper Crust in the research zone. (b) P wave velocity structure of the Middle Crust. (c) P wave velocity structure of the Lower Crust. (d) P wave velocity structure of Lithospheric Mantle. (e) P wave velocity structure of the profile across the BZFZ. The AA’ line shows the location of the profile. The red dotted circles represent the low velocity anomalies in the Middle Crust. The gray belts in the vertical profile are the positions of the faults in our model.

As the average velocies illustrated in Fig 3(a)–3(d), we defined each element by a set of heterogeneous mechanical properties provided by the inversion of the seismic velocity model of HB CRUST 1.0, which is a well-known high-resolution three-dimensional velocity model covering the middle eastern portion of the North China Craton [54]. As depicted in Fig 3(e), several low-velocity anomalies can be seen in the middle crust from the velocity profile across the BZFZ zone.

The above three-dimensional model includes 20 major active faults in the BZFZ and other main NE-trending fault zones within North China. The mechanical properties of the faults (e.g., Young’s modulus and effective viscosity coefficient) are defined to be up to an order of magnitude less than that of the surrounding upper crust [55]. To study the influence of vertical segmentation, we divide each active fault into a locking section and a non-locking section based on the bottom boundary of the upper crust, where sections of different mechanical properties join via a brittle–ductile transition and most big earthquakes in North China happened.

Key in our model, we use the birth–death elements for the locking segments of faults to simulate the re-opening of locking segments after large earthquakes. Note that the stiffness of the birth–death element can be reduced without changing the properties of the other elements.

We applied the boundary conditions based on GPS data and the inverse method [27,30] as follows. The top boundary of the model is set as a free surface; i.e., all stress components are nil. The bottom boundary of the model is horizontal and vertically fixed to zero. The model is characterized by a relatively uniform GPS motion whose rate of movement is generally ESE-oriented [52] (Fig 2). To mimic the GPS motion of the BZFZ, different horizontal strain rates are applied in the four corners of the model based on the GPS observation [52], whereas that of the other boundary nodes are calculated by linear interpolation method (top view in Fig 2).

By comparing the simulation results of different models with various loading of the observation GPS, we analyzed the influence of the uncertainty in different boundary condition and verified the reliable of the forward model. Here we use average dispersion D to measure the fitness between the GPS observation results and calculated results in different models [52].

$$D=\frac{1}{m}\sum_{i=1}^{m}{\left(V_{i}^{obs}-V_{i}^{mod}\right)}^{2}$$ (8)

where D is the average dispersion, m is the total number of GPS observation points, the $V_{i}^{obs}$ observed GPS data at point i, $V_{i}^{mod}$ is the calculated strain rates at point i.

**Fig 4:**
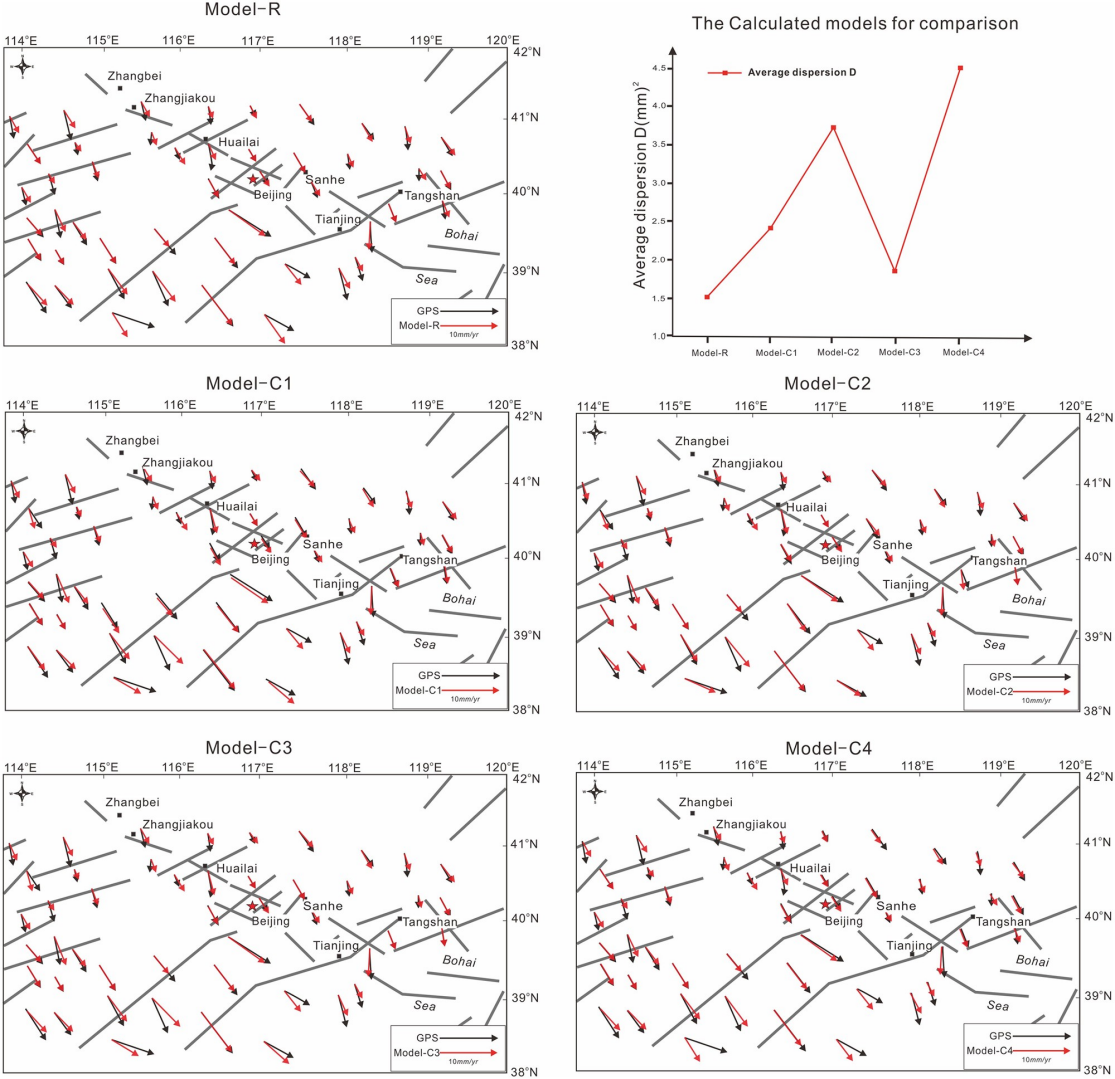
Comparison and average dispersions between GPS(black) and calculated strain. Model-R: the forward reality model based on HBCrust 1.0 and above-mentioned boundary conditions(Fig 2), calculated by linear interpolation method on GPS data of the four corners [52]; Model-C1: boundary conditions calculated by all GPS data in the research zone [52]; Model-C2: boundary conditions calculated by all GPS data in the research zone [53]; Model-C3: boundary conditions calculated by mixed GPS data [52,53]; Model-C4: 10% variation of the boundary strain rates compared to Model-R.

For the models given in Fig 4, the optimal solution to fit the GPS observation data favors the forward model-R (which shows the lowest average dispersion, D = 1.51 mm2/yr2), indicating that the layered rheology structure and boundary conditions are reliable.

### Modeling results and analysis

**Fig 5:**
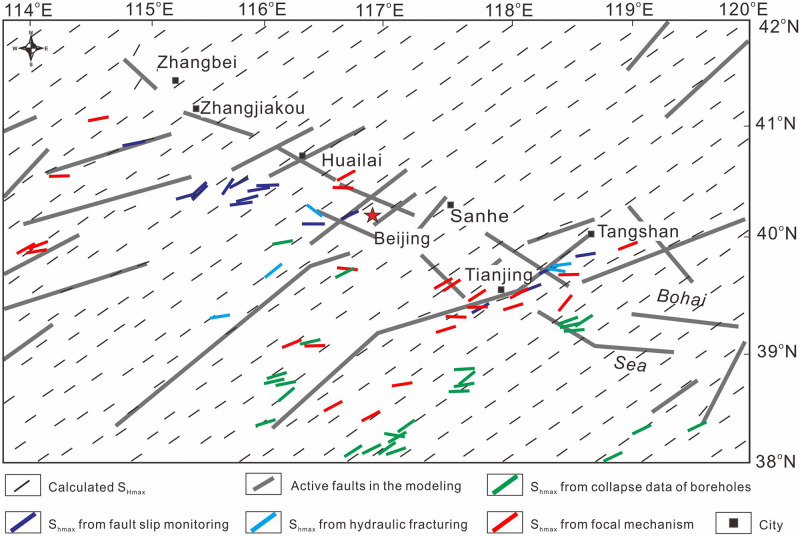
The maximum principal compressive stress trajectory map of the study area compared with the observed data including collapse data of boreholes, fault slip monitoring, hydraulic fracturing and focal mechanism.

**Fig 6:**
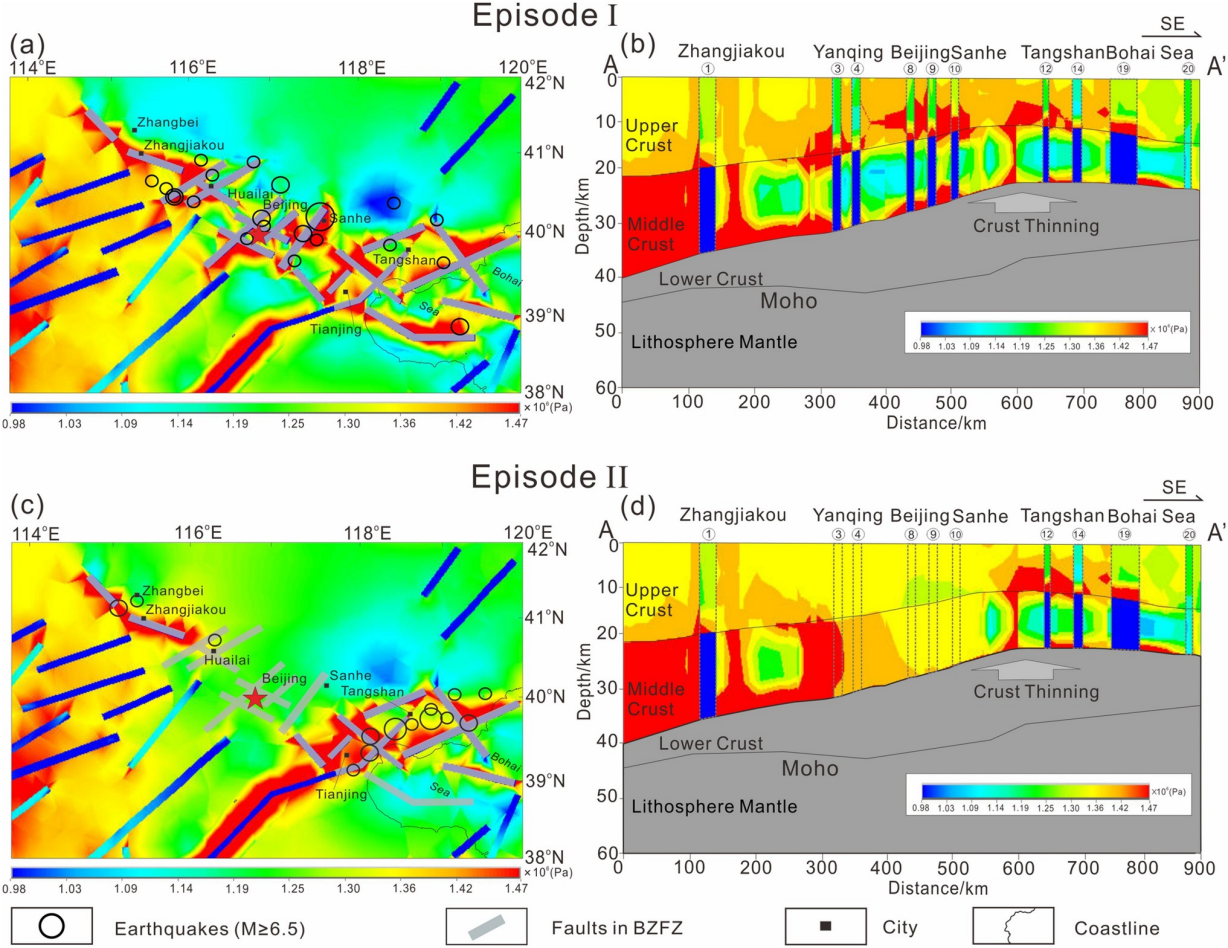
Contours of differential intensity stress (measured in Pa) calculated by the model and historical earthquakes in different episodes in the BZFZ. (a) and (b) represent the differential intensity stress on the plane and profile in episode I. (c) and (d) represent the differential intensity stress on the plane and profile in episode II. Black circles represent big historical earthquakes in each episode.

Given the above three-dimensional viscoelastic heterogeneous model and boundary conditions, we applied a GPS-based displacement field at the edges of the model. We then obtained the maximum principal compressive stress trajectory map of the study area, which we present in Fig 5, and the stress intensity map of each depth plane as well as the trans-BZFZ profile, which have been presented in Fig 6(a)–6(d).

To illustrate the reliability of the calculated stress field, we used horizontal maximum principal compressive stress σHmax from the observed data, which includes collapse data of boreholes, fault slip monitoring, hydraulic fracturing, and the focal mechanism [56] (http://www.eq-icd.cn/webgis/index.htm) for comparison and validation. The calculated trajectories of the horizontal maximum principal compressive stress σHmax fit well with the observed data, which comprised NEE–SWW-trending trajectories, as depicted in Fig 5. The residuals between the observed data and our calculated results are well within the observational errors, indicating the reliability of our calculated stress field [33,41,47,57].

Note that in the first model run of Episode I, all faults in the BZFZ are locked. The evolution of the system in Episode I after 10,000 years is shown in Fig 6(a) as an initial stress field. Here, the stress intensity concentrates near the faults at the bottom boundary of the upper crust as a brittle–ductile transition where most large earthquakes have occurred. Nearly all low-velocity anomalies (as shown in Fig 3(e)) have low-stress intensity both in Episodes I and II (i.e., Fig 6(b) and 6(d)). Most of the stress concentration areas are above the low-velocity anomalies, more specifically near faults 1, 3, 4, 8, 9, 10, and 12; these results are consistent with the conceptual model of low-velocity anomalies in North China, as indicated in Fig 6(b) [36].

During Episode I, higher stress intensities are concentrated within the Huailai-Beijing-Sanhe and Tangshan segments in the southeast regions more than that of the Zhangjiakou segment in the northwest of the BZFZ. One possible reason here is that the southeastern crust is significantly thinner than the northwestern crust, with these crusts divided by the Taihangshan fault zone. The upwelling position of the Moho surface coincides with the area of high stress concentration, as we show in Fig 6(b), where large earthquakes have occurred, including the Sanhe-Pinggu M8.0 earthquake.

In the second model run of Episode II, the locked fault segments where large earthquakes occurred in Episode I are opened up by killing the birth–death elements, which leads to a new adjustment of stress fields and migration of the stress concentration area. The stress intensity in Episode II is shown in Fig 6(c) and 6(d). We observe here that the stress intensity near the faults in the Huailai-Beijing-Sanhe segment where big earthquakes occurred in Episode I (i.e., faults 3, 4, 8, 9, and 10) readjusts to a relatively low level. Further, in Episode II, the stress intensities within the Tangshan and Zhangjiakou segments appear higher than that of Episode I both on the plane and the profile, as indicated in Fig 6(c) and 6(d), respectively, especially along the Tangshan fault and the area near Tianjing within the Tangshan-Cixian-Ninghe Fault Zone, which we show in Fig 6(c).

**Fig 7:**
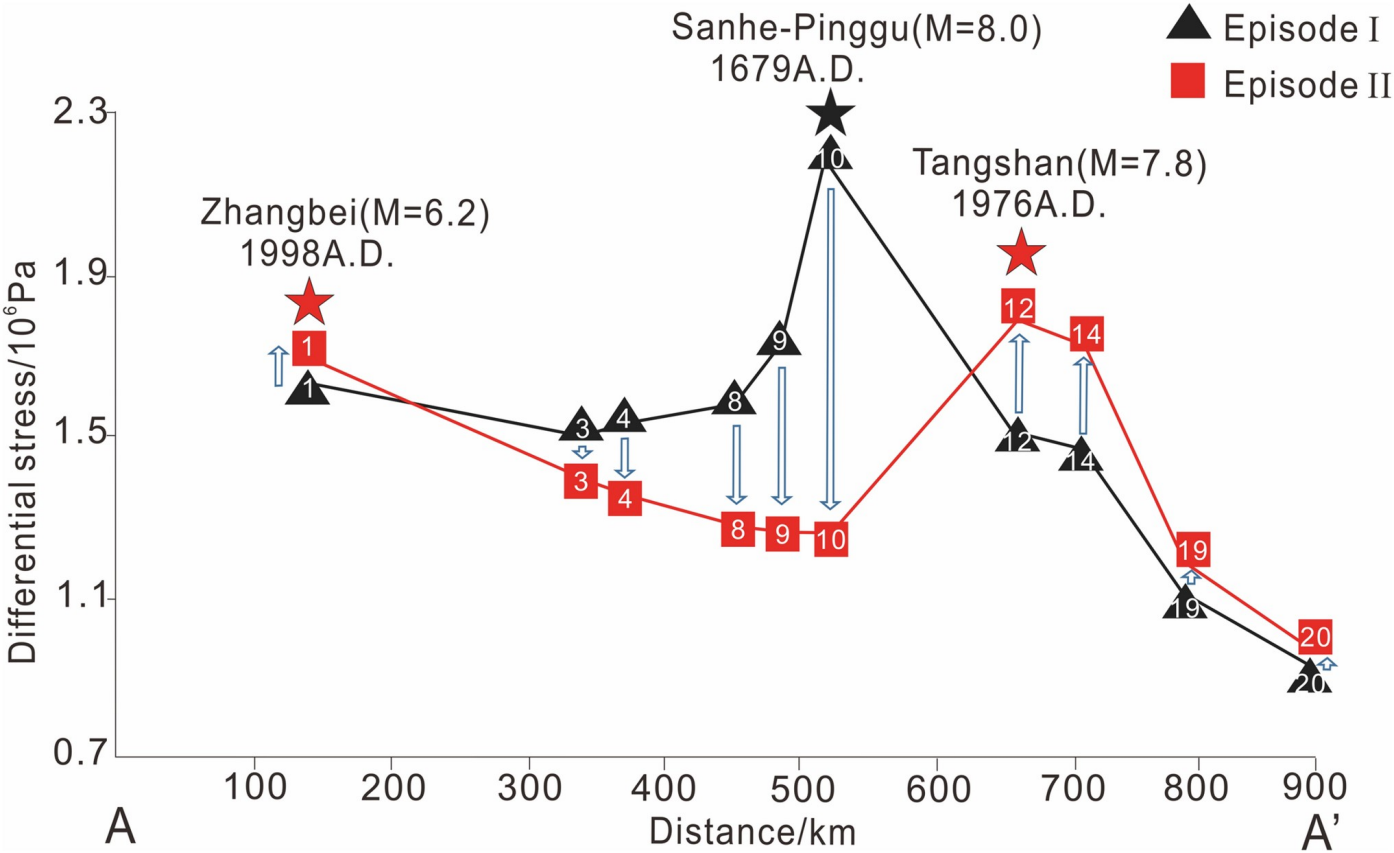
The stress intensity change near the brittle-ductile transition of the main active faults in BZFZ on profile AA’ after episode I. The black triangle represents the stress intensity in episode I. The red triangle stands for the stress intensity in episode II. The stars represent the important historical earthquakes in different segments in the two episodes.

The stress intensity changes near the brittle–ductile transition of the main active faults within the BZFZ after Episode I are shown in Fig 7. We observe here that the largest stress decrease of approximately 1 MPa occurred near the Sanhe-Pinggu fault (i.e., fault 10), where the Sanhe-Pinggu M8.0 earthquake occurred in Episode I. Near other active faults within the Huailai-Beijing-Sanhe segment (i.e., faults 3, 4, 8, and 9), the stress decrease gradually drops from 0.1 to 0.4 MPa as the distance from the Sanhe-Pinggu fault gradually increases. The largest stress increase of approximately 0.2 MPa occurred near the Tangshan fault (i.e., faults 12 and 14), where the Tangshan M7.8 earthquake occurred in 1976. The stress changes near the Zhangbei (i.e., fault 1) and Bohai Sea (i.e., faults 19 and 20) faults, which occurred between the two episodes, were consistently less than 0.05 MPa.

The largest stress increase near the Tangshan fault and the relatively small stress increase near the Zhangbei fault denote that the Tangshan M7.8 earthquake may have been primarily triggered by the Sanhe-Pinggu earthquake in 1679 and other earthquakes in Episode I, whereas according to our modeling, the Zhangbei earthquake in 1998 may not have been triggered by the stress readjustments that occurred after Episode I.

### Conclusions and discussion

In this study, we established a three-dimensional Maxwell viscoelastic model including all active faults within the BZFZ with different spatial patterns, vertical segmentations, and lithospheric heterogeneity to illustrate the mechanism behind large historical earthquake migrations. We calculated the stress field to reveal the underlying mechanisms of earthquake progression and migration on the basis of the geological evidence and previous research conducted in North China.

Firstly, our calculated results reveal that the three main stress concentration region in BZFZ were all located at the transition zones between NW-trending and NE-trending faults, which matched well with distribution of earthquakes. From the perspective of the spatial distribution of the stress concentration area, the NW-trending faults played an important role in triggering earthquakes, but the true seismogenic faults were NE-trending faults.

Our calculated results also reveal a well-matched initiation stress intensity concentration in the main historical earthquake-prone areas within the BZFZ, especially within the Beijing and Tangshan seismic zones.

Further, results of our simulations suggest that the stress decrease within the Huailai-Beijing-Sanhe segment (shown in Fig 7) and the stress increase within the Tangshan segment correspond to the recorded magnitudes of historical earthquakes. Further, the stress increases within the Zhangjiakou segment and faults to the east of the Tangshan region are relatively small. The largest stress decrease occurred near the Sanhe-Pinggu fault, where the largest earthquake within Episode I occurred. Also, the largest stress increase was near the Tangshan fault, where the largest earthquake in Episode II occurred. More specifically, we found the magnitude of the stress decreases to be inversely proportional to the magnitude of the Episode I earthquake, thereby showing that the seismic risk within the BZFZ is time-predictable along similar elapsed times.

One possible reason for the discrepancy of stress localization is the difference in crustal thickness. Because of the destruction of the North China Craton, the crustal thinning to the east of the Taihangshan fault is the strongest. Further, the crustal thickness to the east of the Taihangshan fault is much smaller than that of the west (as indicated in Fig 3(e), which leads to the concentration of stress in the middle and upper crusts. Therefore, in Episode II, the seismic stress primarily migrated east toward the Tangshan Fault Zone.

In addition, the low-velocity body below the Tangshan segment is large and close to the active faults; therefore, the stress intensity increases the most at this point. Further, the low-velocity body below Zhangbei is indeed small and away from the fault; hence, the stress intensity increases at a slow rate. There is no stress concentration observed near the faults without any low-velocity body below, and no large historical earthquakes have occurred near these active faults. The Tangshan earthquake of Episode II is close to the Sanhe–Pinggu earthquake of Episode I, whereas the Zhangbei earthquake is distant from the earthquakes of Episode I, which is also one of the reasons because of which the stress intensity variations differ.

**Fig 8:**
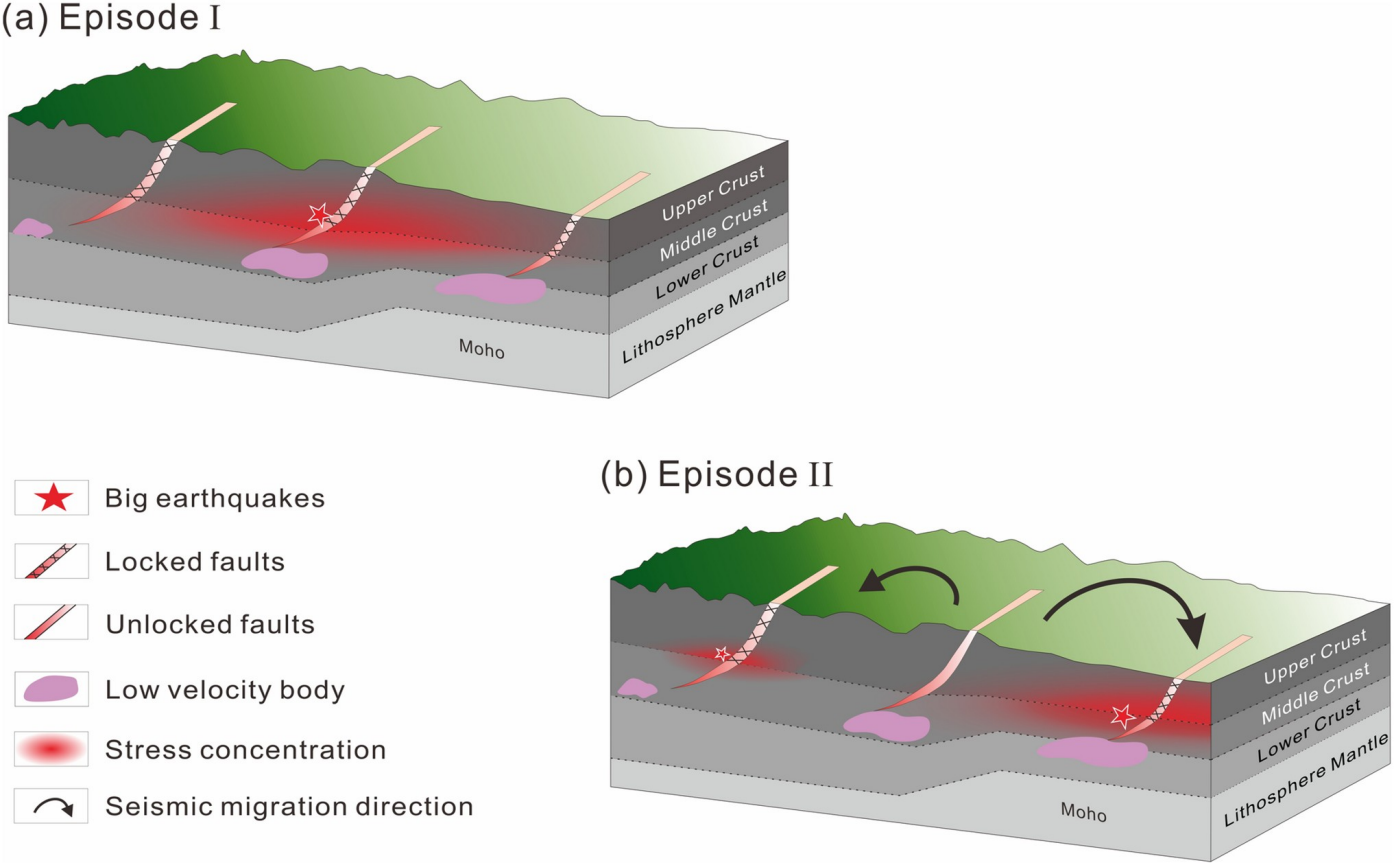
The model of seismic migration of the dynamical mechanisms of the earthquake migration in the BZFZ. In this model, the factors affecting the seismic migration path and intensity mainly include the distance of the triggered active fault from the original fault, the coupling of the active faults, the location, scale of the low velocity anomaly and its distance from the active fault, and the location and scale of the crustal thinning.

Given the above, we propose the model of seismic migration presented in Fig 8 to summarize the dynamic mechanisms of earthquake migration within the BZFZ. The periodic opening-and-locking of active faults is the primary reason for the recurring earthquakes moving in cycles. The factors affecting the seismic migration path and intensity primarily include the distance between the pre-shocking fault and triggered fault, the coupling of the active faults, the location and scale of the low-velocity anomaly, and crustal thinning. The coupling between spatial patterns of pre-existing active faults and the heterogeneity of the lithosphere constrains the intensity of the earthquakes and their migration directions.

Earthquake migration has been well explained by the stress triggering theory. The changes of Coulomb failure stress (CFS) induced by large earthquakes and their triggering effect to subsequent earthquakes have caught wide spread attention in recent years [17,18,46]. The CFS theory changes the idea that the occurrences of large earthquakes are random, independent and irrelevant [58]. The CFS has been well used in North China, to illustrate the mechanisms underlying earthquake migration in North China [13,27,32]. In this research, we want to calculate the tectonic stress intensity changes during the two episodes, and adjustment of regional stress fields caused by the earthquakes thereafter. The tectonic stress change could only explain the intensity of great earthquakes and their positions roughly (Figs 6 and 8). We wish to use CFS method for a precise interpretation of the interaction and migration of earthquakes in North China in our future work.

In conclusion, we found that episodic seismicity and clustering seismic migrations in mid-continents result from the adjustment of regional stress fields after large earthquakes and active fault openings, as well as coupling between deep and shallow geological processes. In our future work, our model of earthquake migration could be considered in the long-term seismic hazard assessment within North China and other mid-continents.

## Supporting information

S1 File Earthquake catalog (Ms5). All earthquakes used in the papers are listed.
(EQT)

S2 File GPS bondary data. Boundary loading data used in the papers are listed.
(TXT)
